# A novel mouse tail lymphedema model for observing lymphatic pump failure during lymphedema development

**DOI:** 10.1038/s41598-019-46797-2

**Published:** 2019-07-18

**Authors:** Michael J. Weiler, Matthew T. Cribb, Zhanna Nepiyushchikh, Tyler S. Nelson, J. Brandon Dixon

**Affiliations:** 1 Parker H. Petit Institute for Bioengineering and Bioscience, Georgia Institute of Technology, Atlanta, GA USA; 2 George W. Woodruff School of Mechanical Engineering, Georgia Institute of Technology, Atlanta, GA USA; 3 Wallace H. Coulter Department of Biomedical Engineering, Georgia Institute of Technology, Atlanta, GA USA

**Keywords:** Cardiovascular diseases, Circulation, Circulation, Cardiovascular diseases, Biomedical engineering

## Abstract

It has been suggested that many forms of secondary lymphedema in humans are driven by a progressive loss of lymphatic pump function after an initial risk-inducing event. However, the link between pump failure and disease progression has remained elusive due to experimental challenges in the clinical setting and a lack of adequate animal models. Using a novel surgical model of lymphatic injury, we track the adaptation and functional decline of the lymphatic network in response to surgery. This model mimics the histological hallmarks of the typical mouse tail lymphedema model while leaving an intact collecting vessel for analysis of functional changes during disease progression. Lymphatic function in the intact collecting vessel negatively correlated with swelling, while a loss of pumping pressure generation remained even after resolution of swelling. By using this model to study the role of obesity in lymphedema development, we show that obesity exacerbates acquired lymphatic pump failure following lymphatic injury, suggesting one mechanism through which obesity may worsen lymphedema. This lymphatic injury model will allow for future studies investigating the molecular mechanisms leading to lymphedema development.

## Introduction

More than 130 million individuals worldwide suffer from lymphedema, a chronic disease that presents with the accumulation of fluid, proteins, and adipocytes in the interstitium, resulting in a drastic enlargement of the affected limb^1,2^. The most common form is secondary (acquired) lymphedema, which results from the disruption of previously normal lymphatic networks, usually through injury or surgical procedure^3^. In developing countries, most secondary lymphedema cases occur due to lymphatic filariasis from parasitic infections. In developed countries, the two main contributors to secondary lymphedema are surgical and radiotherapeutic cancer interventions, with breast-cancer related lymphedema being one of the most encountered and studied forms^4^. Surprisingly, most secondary lymphedema cases present 1–3 years or more after the insulting event, rather than immediately following the trauma^1^, and it is this latency period that makes lymphedema very difficult to diagnose early and treat effectively.

Despite the prevalence of lymphedema, there is very little known about the exact mechanisms of disease progression, especially as it relates to lymphatic transport function and the progressive loss thereof. In particular, it remains unclear how the lymphatic collectors, which transport the bulk of lymph and are the main functional units of the lymphatic system, respond to changes in mechanical loading and the inflammatory milieu during the post-trauma period to contribute to the onset of lymphedema. The latency period commonly associated with lymphedema motivates a hypothesis of remodeling-induced lymphatic pump failure, which has begun to gather support in the lymphedema literature^3^, and has been hinted at in certain animal models of lymphatic injury^4–6^. Inflammation, in particular, has been shown to contribute to the degree of swelling in lymphedema^7^ and may influence the onset and perpetuation of reduced pump function^3^. It is well documented that the injury or surgical wound space forms into scar tissue, which subsequently limits new lymphatic vessel growth in the space and hampers interstitial fluid flows^8,9^. There is also evidence that an inability of the lymphatic system to adequately regenerate during normal wound repair may predispose the tissue to swell during secondary lymphedema^9^. However, it has also been shown that accumulating lymph may experience compensatory drainage through a reduced number of surviving local lymphatics, rerouted lymphatics that bypass the obstructive tissue, or lymphovenous communications^10,11^.

Since lymphatic flow is driven primarily through the contractility of collecting lymphatic vessels, the ability to quantify lymphatic pump function through the imaging of functional lymphatic transport in the context of early lymphedema onset would greatly enhance our understanding of the disease progression and would foster the development of diagnostic technologies that could directly detect the underlying defect^12^. We hypothesize that lymphedema is associated with a progressive loss of lymphatic pump function, which can be visualized and subsequently quantified with near-infrared (NIR) imaging, thus affording novel insights into the disease cascade. However, the paucity of robust animal models of lymphedema has limited study of the disease. The most common model in the literature is the rodent tail model, which involves severing all initial and collecting vessels in the tail to induce a lymphedema cascade^13–15^. We will refer to this model as the double vessel ligation model. Other reported models include the dog hindlimb^16^, sheep hindlimb^17^, rabbit ear^16^, and rodent limb^3,18^. These models have been instrumental in our early understanding of lymphedema pathogenesis and the resulting changes in the interstitium, but in each of these models all the collecting vessels draining the tissue space of interest have been severed, and the analysis has focused exclusively on morphological changes occurring in the dermal layer and the resulting lymphangiogenic response that occurs in the initial lymphatics. No model currently exists that allows the preservation of functional collecting vessel trunks, and no study to date has investigated the changes in collecting vessel function associated with lymphedema.

Therefore, we have developed a new rodent tail model of lymphedema allowing for selective preservation of individual lymphatic collectors to track changes in the collecting vessel transport metrics over time using NIR lymphatic imaging. The goal was to develop a model that exhibits similar pathological progression in the skin as the classic rodent tail model of lymphedema while preserving a single intact collecting vessel trunk for functional time-course analysis. This new model provides the first opportunity to study the link between lymphedema progression and lymphatic collecting vessel function and helps to generate new insights into the potential mechanisms of lymphatic failure during disease progression. In this study we have performed the first investigation of the time-course change in collecting vessel transport capacity in the context of post-procedure lymphedema progression, with an emphasis on the examination and characterization of collecting vessel remodeling events associated with changes in pump function.

To show additional utility in the use of this model as a tool for studying the development of lymphedema, we tested the surgical model in mouse fed a high-fat diet (HFD) to induce obesity. One of the strongest associations with the onset of lymphatic dysfunction is obesity. Recent clinical studies have made it abundantly clear that pre-surgical BMI strongly correlates with post-operative development of lymphedema in breast cancer patients. However, the cause of this correlation is unknown, and it is unclear how obesity impacts lymphatic function during lymphedema development. Our novel single vessel ligation lymphedema model allows for analysis of functional changes in the intact lymphatic vessel following lymphatic ligation in the context of obesity.

## Results

### Single vessel ligation lymphedema model produces differential swelling response

**Figure 1:**
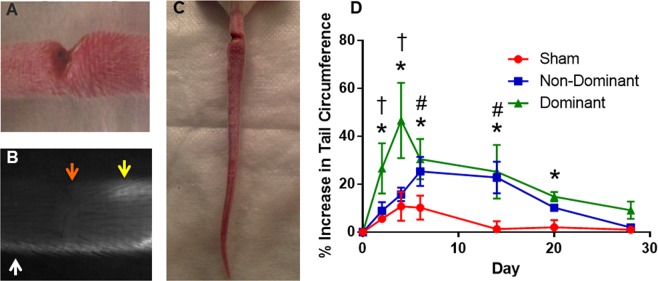
Tail ligation model and swelling cascade. (**A**) Example incision and cauterization of tail to sever right collecting vessel and approximately 80% of initial lymphatic network. (**B**) Example NIR image showing the incision (orange arrow), the right collecting vessel severed, and the left collecting vessel intact (white arrow). (**C**) Example swollen tail at one-week time point. (**D**) Plot of tail swelling of three experimental groups. Both ligation groups produced increased swelling compared to the sham between weeks 1 and 2. The dominant ligation also produced increased swelling compared to the sham at days 2 and 4 and week 3. The dominant ligation produced significantly increased swelling compared to the nondominant ligation at days 2 and 4. *Dom significant compared to sham, t = Dom significant compared to NonDom, ^#^NonDom significant compared to sham.

Three different surgical models were used to determine the functional response of the collecting lymphatic vessels to various degrees of injury: (1) a vessel ligation group in which the dominant vessel was ligated, (2) a vessel ligation group in which the nondominant vessel was ligated, and (3) a sham control group in which both collecting vessels were left intact. Collecting vessels exist on both sides of the mouse tail and the dominant vessel was identified through NIR imaging as the vessel which first showed fluorescence within the imaging area as has been described in detail previously in the rat^19^. Tail swelling significantly increased as a result of collecting lymphatic vessel ligation compared to the sham group, and ligating the dominant vessel produced significantly more swelling than ligation of the non-dominant vessel (Fig. 1D). Dominant ligation swelling peaked at 45% on day 4, while non-dominant ligation swelling reached a maximum of 28% swelling on day 14. Both ligation groups returned to normal circumference values on or soon after day 28, which is similar to the kinetics of swelling resolution published in other studies in the mouse tail, although the magnitudes of peak swelling are substantially less. The sham control group in which a skin incision was made but no collecting lymphatics were damaged, experienced a mild swelling response with peak swelling of 16% on day 4 and full resolution by day 14.

### Single vessel ligation lymphedema model mimics histological hallmarks of typical mouse tail lymphedema model

**Figure 2:**
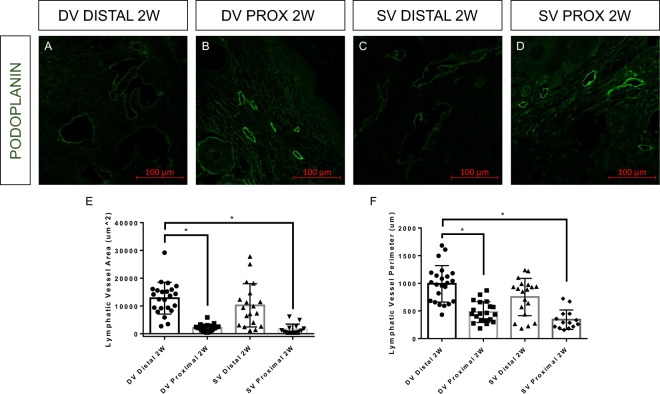
Single and double vessel lymphatic ligation result in lymphatic hyperplasia following surgery. (**A**–**D**) Mouse tail sections harvested distal and proximal to the site of lymphatic injury at two weeks following surgery were stained for podoplanin to determine lymphatic vessel area and perimeter. Lymphatic vessel area and perimeter in double vessel (DV) distal sections at two weeks were significantly increased compared to DV proximal sections at two weeks and single vessel (SV) proximal sections at two weeks. There was no significant difference between SV and DV distal sections at two weeks. (**E**,**F**) Quantification of lymphatic vessel area and perimeter from sections shown in (**A**–**D**) (n = 2 per group, at least 6 hpf per section).

Tail sections were harvested at two weeks following lymphatic injury in the dominant vessel ligation group. Tail sections were also harvested at two weeks in mice that underwent double vesssel ligation. Sections were harvested distal and proximal to the site of injury. These sections were paraffin-embedded and sliced for immunohistochemical analysis. Immunohistochemistry (IHC) was used to stain for podoplanin, a lymphatic marker (Fig. 2A–D). Sections distal to the wound in the double vessel ligation group showed significantly increased lymphatic vessel area and perimeter compared to sections proximal to the wound in both the single and double vessel ligation groups (Fig. 2E,F). However, sections distal to the wound in the double vessel ligation group showed no differences in lymphatic vessel area and perimeter compared to sections distal to the wound in the single vessel ligation group. These results show that the single vessel ligation lymphedema model mimics the histopathological response seen in the double vessel ligation lymphedema model, specifically lymphatic hyperplasia, at the timepoint of peak swelling.

### Vessel contraction creates packets

**Figure 3:**
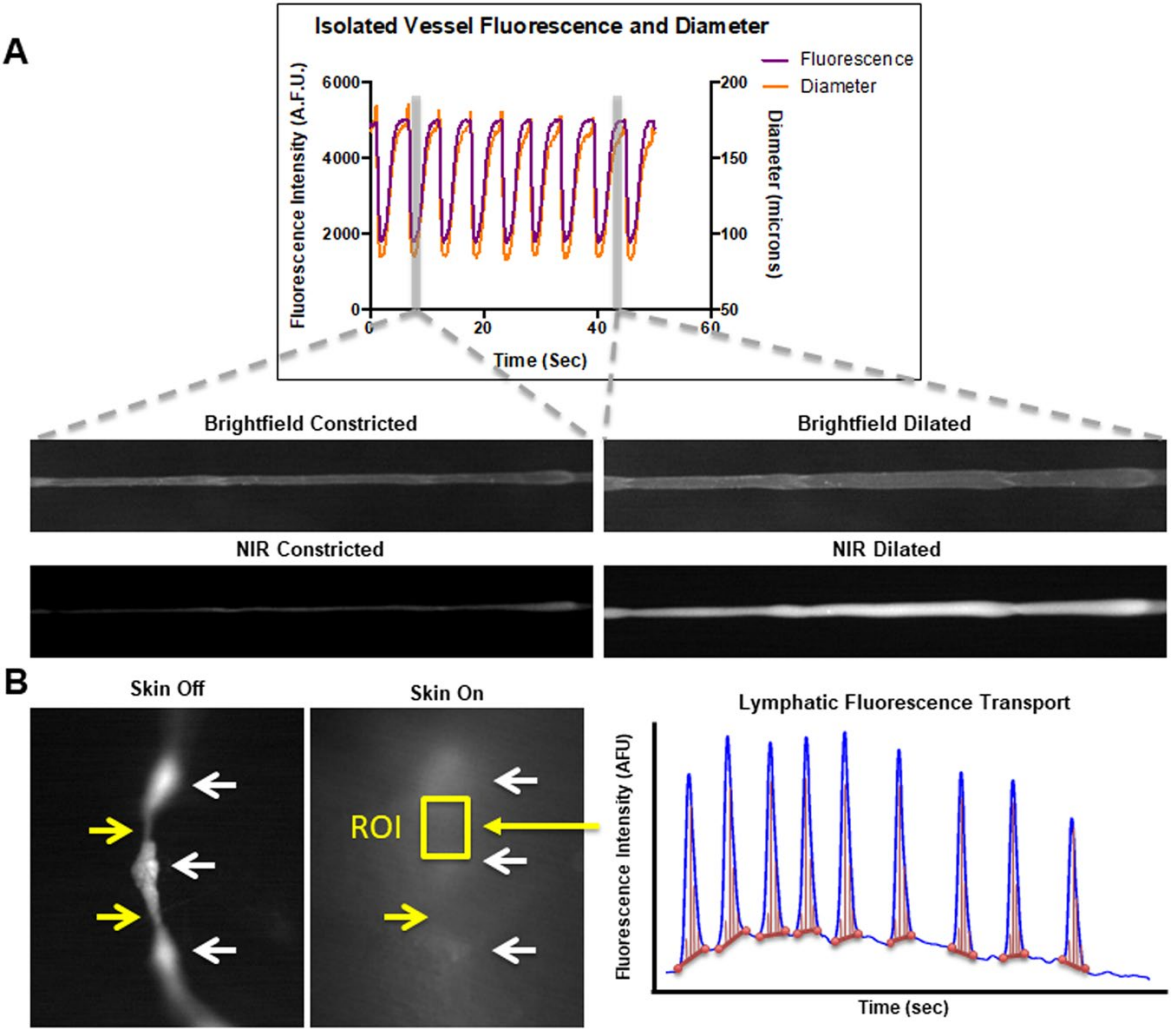
NIR Fluorescence Transport Analysis. (**A**) Example plot and representative images of NIR and brightfield imaging of an isolated vessel. Contraction dynamics are in sync with fluorescence intensity indicating that packets are created by the contraction and dilation of the vessels. (**B**) Example NIR image with the skin off and the skin on. The image on the left shows NIR dye flowing through the collecting vessel of a rat forelimb with the skin removed. Dye can be seen pooling in the sinus regions of the vessel, but the entire length of vessel is fluorescent. The image on the right shows the same vessel with the skin on. The fluorescent signal is much weaker and more scattered, and fluorescence is only directly visible in the areas corresponding to the sinus regions, where more fluorophore accumulates. White arrows indicate the three sinus regions in each image, and the yellow arrows indicate the tubular section between the sinuses in which NIR fluorescent transport analysis is performed. The plot shows an example of fluorescence over time in a region of interest in a tubular section of a collecting vessel. The packet integral was calculated by first defining the spikes in intensity corresponding to packets and then integrating the signal in these regions. The red circles and horizontal lines represent the packet minimum intensity while the red vertical lines represent the integral over the packet. Lymphatic fluorescence transport was calculated by summing the packet integrals and normalizing by time.

In order to better characterize NIR imaging of lymphatic function, an *ex vivo* experiment using isolated rat mesenteric vessels was performed. Brightfield and NIR images were taken together of contracting isolated lymphatic collecting vessels to compare vessel functional dynamics with function as measured by NIR imaging. The isolated vessel setup revealed that vessel contraction caused the appearance of packets in NIR imaging (Fig. 3A), which was also recently confirmed in another report^20^. As the vessel constricts below a certain threshold, the amount of fluorophore in the local region is too low to be detected with NIR imaging. Comparing vessel contraction dynamics under brightfield to the appearance of packets in NIR imaging reveals that the two phenomena are perfectly in sync. After showing that NIR imaging of lymphatic function matched *ex vivo* contractions of collecting vessels, an experiment was performed to better characterize *in vivo* contractions using NIR imaging. Images were taken in the mouse forelimb after fluorophore injection in the footpad with the skin on and the skin off to determine how the skin affects the appearance of packets *in vivo*. This experiment revealed that areas of NIR dye stagnation occur immediately downstream (proximal) from the valve regions in the sinus of the lymphangions (Fig. 3B). The sinuses are almost always visible under NIR, but the tubular regions, which are the segments of the vessels that produce the largest contractions in isolated vessel preparations, are only visible when it is dilated above a certain threshold. Therefore the peak-to-peak variation in fluorescence amplitude is comparable to the contraction amplitude measured in isolated vessel experiments while lymphatic fluorescence transport is effectively a measurement of fractional pump flow, which is similar to the pumping score developed by Chong *et al*.^20^.

### Lymphatic dysfunction as measured by packet transport correlates with increased swelling under a variety of conditions

**Figure 4:**
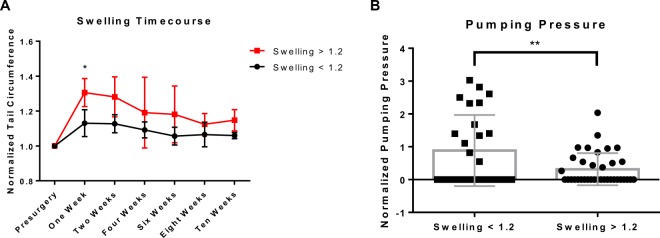
Lymphatic pumping dysfunction is maintained after significant swelling. (**A**) Plot shows the normalized tail circumference measured over ten weeks postsurgery between the two different swelling classifications. There is a significant difference in swelling between the two groups at one-week postsurgery (p = 0.0013). (**B**) Plot shows normalized pumping pressure compared between two different swelling classifications (less than or greater than 20% swelling). The normalized pumping pressure measured in mice with less than 20% swelling is shown to be significantly higher than in mice with greater than 20% swelling (p = 0.0069). Values are normalized to presurgery baseline. *Significant between swelling groups, p < 0.05; **significant between swelling groups, p < 0.01.

**Figure 5:**
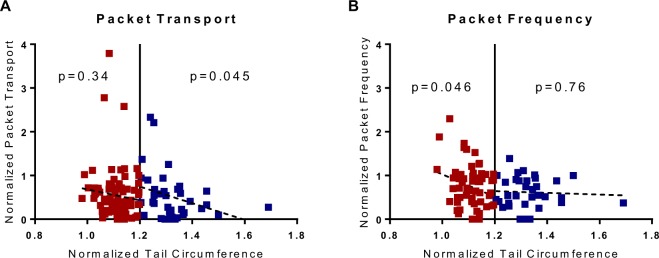
Lymphatic collecting vessel function negatively correlates with swelling. (**A**,**B**) Plots show the correlation between normalized tail circumference and normalized packet transport (**A**) or normalized packet frequency (**B**) for two different swelling classifications (less than or greater than 20% swelling). Values are normalized to presurgery baseline. Data where swelling is less than 20% is shown in red while data where swelling is greater than 20% is shown in blue. There is a significant negative correlation between normalized packet transport and tail circumference when swelling is greater than 20% (**A**: R^2^ = 0.11, p = 0.045), and a significant negative correlation between normalized packet frequency and tail circumference when swelling is less than 20% (**B**: R^2^ = 0.07, p = 0.046).

The surgical model of lymphedema proposed here results in a differential swelling response due to the collecting vessel that is left intact. In order to test whether the extent of swelling correlated with lymphatic function as measured by NIR fluorescence imaging, a heterogeneous group of mice were included in this study. This heterogeneity included variation in gender, strain (BALB/c and C57BL/6), and diet (chow and high fat). To compare mice with little swelling to those with significant swelling, two groups were categorized: mice with less than 20 percent increase in maximum circumference and those with more than 20 percent increase in maximum circumference. In humans, lymphedema is often characterized as a difference of 10 percent in volume between limbs; since this mouse model of lymphedema results in larger relative swelling, 20 percent was chosen as the threshold. As a point of reference, swelling in the double ligation model is typically around a 40–50% increase in max circumference at the peak of swelling. Mice that swelled greater than 20 percent at any timepoint over the course of the ten-week study showed significantly different swelling responses in the week after surgery compared to mice that never swelled greater than 20 percent (Fig. 4A), suggesting that it is either the surgery itself or the immediate response to surgery that drives the severity of swelling in this animal model. This difference in swelling response was not significant in later weeks. There was a significant difference in lymphatic pumping pressure between the two swelling groups (p = 0.0069), suggesting differences in lymphatic function correlate with changes in swelling (Fig. 4B). Using the 20 percent swelling threshold, it was found that swelling negatively correlated with packet transport in mice with increased swelling (n = 14, p = 0.045), while there was no correlation between swelling and packet transport in mice with less swelling (Fig. 5A). It was also found that swelling did not correlate with packet frequency in mice with increased swelling, while there was a negative correlation between swelling and frequency in mice with less swelling (n = 4, p = 0.046) (Fig. 5B). Although it is difficult to say whether the swelling response leads to lymphatic dysfunction or these decreases in lymphatic function drive swelling, these results show that the extent of swelling severity is closely tied to changes in lymphatic function. The differences in the effect of swelling on packet frequency and packet transport show that changes in packet frequency do not necessarily reflect changes in overall transport capacity.

### Reductions in lymphatic transport correlate with disease progression

**Figure 6:**
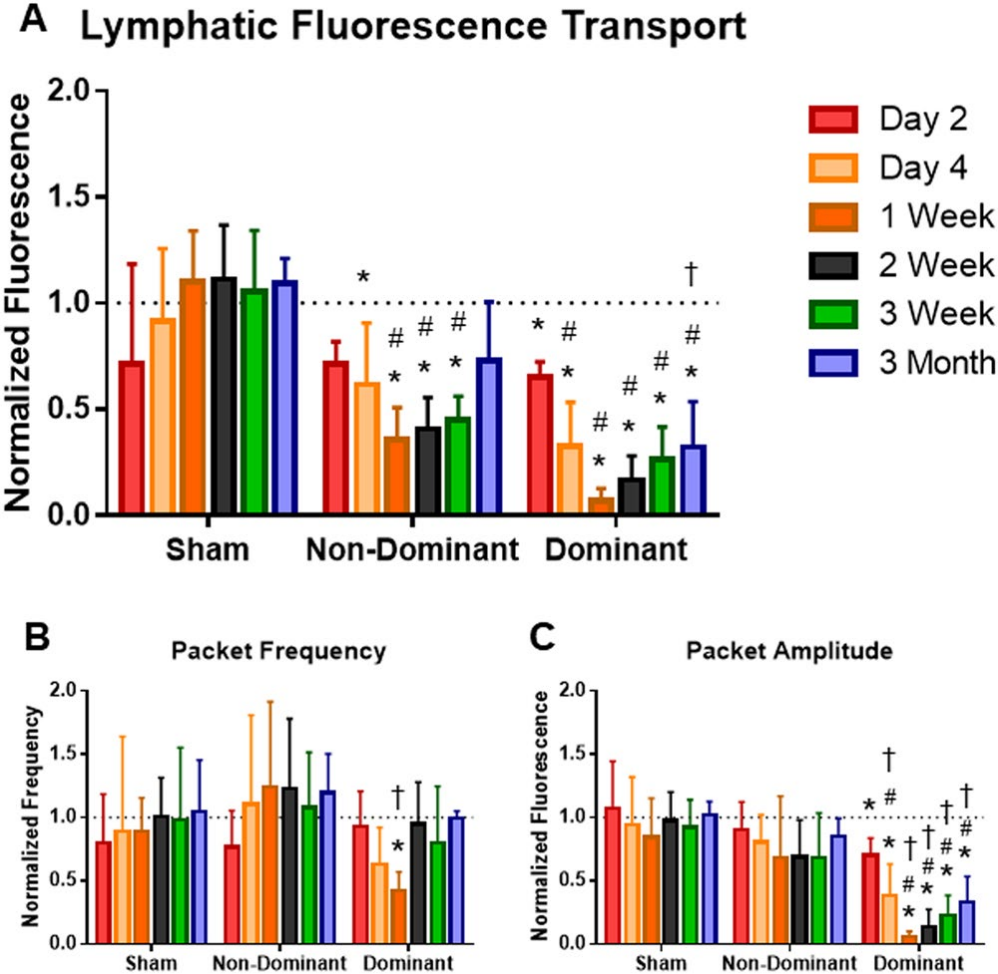
Lymphatic fluorescence transport over time. (**A**) Plot showing normalized lymphatic fluorescence transport for three experimental conditions. Values are normalized to presurgery baseline. Both ligation groups produced significantly reduced transport compared to the sham between weeks 1 to 3. The dominant ligation also produced significantly reduced transport compared to the sham at day 4 and month 3. The dominant ligation produced significantly reduced transport compared to the nondominant ligation at month 3. (**B**) Packet frequency in the dominant ligation group was significantly reduced compared to baseline and the nondominant ligation at week 1. (**C**) Packet amplitude in the dominant ligation group was significantly reduced compared to baseline at all time points, and significantly reduced compared to sham and the nondominant ligation at all time points except for day 2. *Significant compared to baseline, ^#^significant compared to sham, t = significant compared to NonDom.

To analyze how lymphatic transport in the intact collecting vessel changed over time during lymphedema progression, we measured changes in lymphatic transport metrics in a group of mice where swelling increased above 20 percent. Normalized lymphatic fluorescence transport was significantly reduced in both the dominant and non-dominant ligation cases as compared to baseline presurgical values and the sham control group (Fig. 6A). Specifically, the nondominant group produced significantly reduced transport values as compared to baseline at day 4 and weeks 1, 2, and 3 and compared to the sham group at weeks 1, 2, and 3. The dominant group produced significantly reduced transport values relative to baseline at all time points and relative to the sham group at day 4, weeks 1, 2, and 3 and month 3. The sham group did not produce significantly altered transport values at any time points compared to baseline.

Lymphatic transport decreased to a minimum value at the 1-week time point in the dominant (8% of healthy transport) and non-dominant cases (35% of healthy transport) before beginning to improve at later time points. At the three-month time point transport in the dominant ligation was significantly reduced compared to both the nondominant ligation and the sham, while transport in the non-dominant ligation was not significantly reduced from baseline healthy values.

Packet frequency in the dominant ligation group was significantly reduced compared to both baseline and the nondominant ligation at week 1, but was not significantly reduced compared to the sham group, as is shown in Fig. 6B. Packet amplitude in the dominant ligation group was significantly reduced compared to baseline at all time points and significantly reduced compared to both the nondominant ligation and sham at all time points except for day 2 (Fig. 6C), suggesting that while contraction frequency returned to normal values, the vessel exhibited sustained deficiencies in the ejection fraction achieved during each contraction.

### Lymphatic vasculature remodels after lymphedema

**Figure 7:**
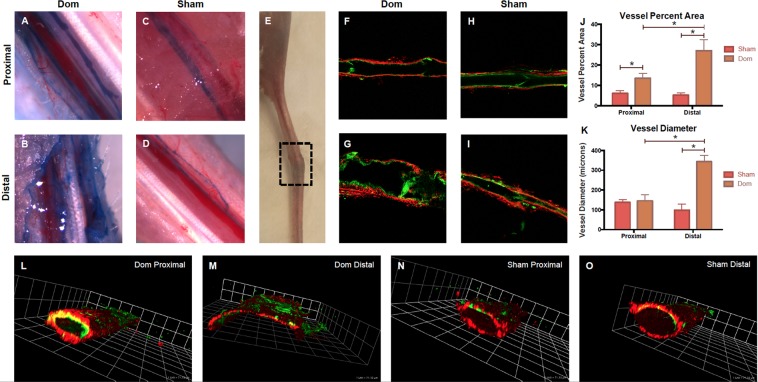
Vessel remodeling at 1-month time point. Unsevered, nondominant collecting trunks were imaged via Evan’s Blue injection and removal of a skin flap (**A**–**D**) and multiphoton microscopy (**F**–**I**). Collecting vessel segments on the proximal side of the ligation site showed a normal phenotype and no evidence of remodeling (**A**,**C**). Collecting vessel segments on the distal side of the ligation site in the Dom group showed extensive lymphangiogenesis and evidence of tortuous vessels via Evan’s Blue (**B**), while the sham group did not show any change on the distal side. (**D**) Swelling at the wound site had resolved one month after surgery, and collecting vessels were isolated from both the distal and proximal side of the wound. (**E**) Multiphoton imaging of collagen (red) and elastin (green) of the distal vessel segments revealed abnormal vessel wall architecture, the possible loss of valve integrity, and apparent lumenal distention in the Dom group. (**G**) The proximal side of the Dom group and both sides of the Sham group showed normal architecture. (**F**,**H**,**I**) Vessel percent area and vessel diameter was significantly elevated in the Dom group on the distal side of the ligation compared to the proximal side and the Sham group. (**J**,**K**) Vessel percent area was measured as the percent area covered by Evan’s blue dye, showing the pronounced leakiness in the vessel distal to the wound in the Dom group. Representative 3D reconstructions of the multiphoton images clearly show an enlargement of the vessel lumen in the remodeled collecting vessels distal to the wound (diameter ranging from 300–400 um) compared to vessels from the proximal location and to the sham vessels (100–200 um) (**L**–**O**).

Microscopy of the unsevered nondominant collecting vessel revealed extensive remodeling on the distal side of the ligation site in the dominant ligation group. Nondominant collecting vessel segments on the proximal side of the ligation site showed a normal morphology and no evidence of gross remodeling (Fig. 7A,C,E). Nondominant collecting vessel segments on the distal side of the ligation site showed extensive lymphangiogenesis and evidence of tortuous vessels via Evan’s Blue (Fig. 7B). Collecting vessels were carefully dissected and mounted for multiphoton imaging using second harmonic generations to image collagen microstructure (red) and wavelength-specific autofluorescence to image elastin (green). Imaging of the distal non-dominant vessel segments revealed abnormal vessel wall architecture, the possible loss of valve integrity, and apparent lumenal distention (Fig. 7G) compared to the sham vessels (7H-I) and the proximal non-dominant vessel (Fig. 7F). 3D reconstructions of the image stacks in Volocity revealed a 2–3 fold enlarged lumen and irregular geometry in the remodeled vessels (Fig. 7L–O).

**Figure 8:**
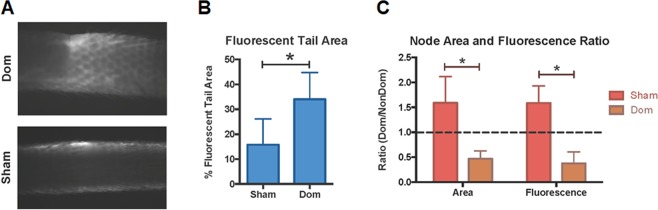
Vessel network remodeling at the 3-month time point. (**A**) Representative images of transport of NIR dye through the tail after injection in the dominant ligation group (top) and the sham group (bottom). (**B**) Fluorescent tail area was significantly larger in the dominant ligation compared to the sham. (**C**) Lymph node area and fluorescence ratios significantly decreased between the sham and dominant ligation groups, indicating a shift towards comparatively larger and more fluorescent nondominant nodes in the dominant ligation group.

At the three-month time point swelling in all conditions completely subsided, but several factors suggest that the dominant ligation group experienced substantial remodeling in lymphatic function compared to the sham control. Percent positive fluorescent tail area, a measure of the relative distribution of lymphatic transport between the collecting vessels and the initial capillary network, was significantly increased in the dominant ligation (Fig. 8A,B). Lymph nodes were also harvested at the three-month time point and their size was measured directly. In the sham, the dominant lymph node was significantly larger and retained significantly more fluorescence than the non-dominant node, but this result was reversed in the dominant ligation (Fig. 8C).

### Obesity exacerbates lymphatic pump dysfunction during lymphedema progression and resolution

**Figure 9:**
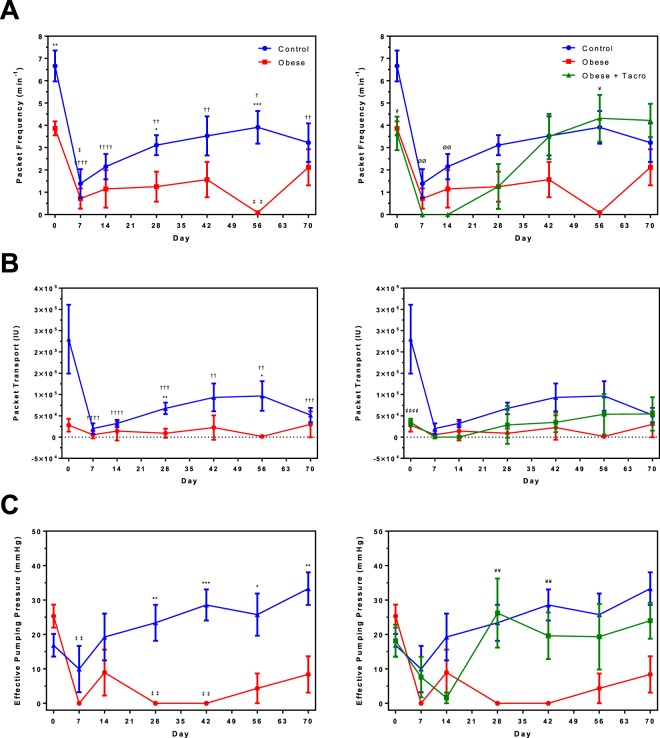
Functional measurements, obtained via NIR imaging, indicate that obesity adversely impacts the functional response of the intact lymphatic vessel following surgery while tacrolimus protects against declines in collecting lymphatic function. (**A**) Packet frequency exhibited significant differences between control and obese groups. Tacrolimus treatment for a subset of obese mice increased lymphatic packet frequency to levels similar to control with significant differences between the obese group manifesting at 56-days post-surgery. (**B**) Packet transport was used to evaluate fluorescence transport driven by intrinsic lymphatic contractions. Tacrolimus treatment was not found to significantly improve lymphatic packet transport. (**C**) Effective pumping pressure was used to evaluate the maximal pressure generated by the collecting vessel to overcome occlusion. Tacrolimus treatment protected against the declines in pumping pressure caused by obesity with significant differences from the untreated group being observed at days 28 and 42. Tacrolimus treatment began at the 14-day time point. Error bars represent SEM. (n = 8, control) (n = 6, obese) (n = 5, obese + tacro) (*denotes significant difference between control and obese; ^†^denotes significant difference between control value and the baseline pre-surgical control value; ^‡^denotes significant difference between obese value and the baseline pre-surgical obese value, ^¢^denotes significant difference between obese + tacro and control; ^¥^denotes significant difference between obese + tacro and obese; ^Ø^denotes significant difference between obese + tacro and baseline day 0 value, p < 0.05).

We utilized our novel single vessel ligation lymphedema model to assess the functional response of the intact lymphatic vessel during lymphedema progression and resolution in a mouse model of obesity. Mice were placed on a high-fat diet (HFD) for twenty weeks prior to surgery to induce obesity. Functional measurements indicate that obesity adversely impacts the functional response of the intact lymphatic vessel following surgery. Packet frequency was found to be significantly decreased at multiple time points in obese animals compared to control (Fig. 9A). Pre-surgical values for packet frequency were significantly different between control and obese at 6.7 ± 0.7 min^−1^ vs 3.9 ± 0.3 min^−1^. Following surgery, the average packet frequency of the intact lymphatic vessel acutely declines at day 7 by roughly 80% of the pre-surgical value for both control and obese animals. Obese animals have significantly lower absolute packet frequency compared to controls at days 0, 28, and 56 (Fig. 9A). Similar trends are observed in packet transport. Control animals had greater impairment of lymphatic packet transport caused by the surgery when compared to pre-surgical baseline values that subsequently persisted throughout the course of the study. However, obese animals exhibited significantly lower absolute values for packet transport compared to controls at the 28 and 56-day time points (Fig. 9B). Lymphatic pumping pressure was found to present striking differences. Despite pre-surgical pumping pressure values being relatively similar between control and obese, obese animals displayed profound decreases following the surgery. Specifically, at the 28-day time point, obese animals exhibited no detectable pumping pressure compared to control with values of 0.0 ± 0.0 mmHg vs 23.4 ± 5.2 mmHg respectively. A similar difference was observed at the 42-day time point. Interestingly, at the end of the study, 70 days after the initial surgery, obese animals still exhibited significant latent defects in lymphatic pumping pressure compared to controls (8.4 ± 5.3 mmHg vs 33.3 ± 4.8 mmHg) that were not otherwise evident pre-surgery (Fig. 9C). Taken together these results indicate that obesity adversely alters the function of the intact lymphatic vessel following surgery. When comparing the absolute differences between the control and obese groups, it becomes apparent that obesity results in lowered contractile frequency, actively driven transport, and pumping pressure of the collecting lymphatics at multiple time points. The most profound differences were observed in the persistently lowered lymphatic pumping pressure, suggesting impairments to the force generating abilities of the lymphangions or failure of the valves to prevent backflow.

### Inhibition of T-cell differentiation with tacrolimus protects against the functional declines caused by obesity during lymphedema

To determine the extent that T-cells are mediating the differences in lymphatic function caused by obesity during lymphedema, we pharmacologically prevented local T-cell activation and differentiation by treatment with a calcineurin inhibitor called tacrolimus. A subset of obese animals (n = 5) were treated via topical application of tacrolimus ointment twice daily beginning 14-days after induction of lymphedema. Results from these experiments show that tacrolimus does not appear to differentially impact the resolution of tail swelling following treatment; however, this could be because the obese + tacrolimus treated experimental group swelled, on average, more than the obese group, despite undergoing identical experimental protocols up until treatment with tacrolimus. In any case, the tail volumes of both treated and untreated animals resolve when given enough time, similar to observations in non-obese mice.

Our NIR functional imaging techniques were used to assess the response of the intact collecting lymphatic vessel to tacrolimus treatment. Tacrolimus was found to positively impact the packet frequency of the intact lymphatic vessel in a differential manner from the untreated group. Specifically, tacrolimus treatment appeared to aid the recovery of packet frequency, significantly increasing to 4.3 ± 1.1 min^−1^ vs 0.0 ± 0.0 min^−1^ in the untreated obese animals at day 56. In fact, the tacrolimus treated animals achieved day 70 values of packet frequency comparable to chow fed controls, despite having significantly lower baseline values than controls (Fig. 9A). Similar trends were observed with regards to packet transport; however, these trends were not statistically different (Fig. 9B). One of the more striking alterations caused by tacrolimus treatment was the recovery observed in lymphatic pumping pressure. Specifically, the tacrolimus treatment improved lymphatic pumping pressure of obese animals to levels similar to chow fed controls by day 28. Given that the untreated obese animals had no detectable effective pumping pressure at days 28 and 42, tacrolimus appears to facilitate a striking improvement in the pressure generating capabilities of the lymphatics (Fig. 9C). Taken together, these results suggest that pharmacological treatment with tacrolimus attenuates the decline in the active contractile response of the intact lymphatic vessel associated with obesity during lymphedema.

## Discussion

In this study we have demonstrated the first direct link between swelling and the progressive loss of lymphatic transport function during the lymphedema disease cascade *in vivo*. The results show that there is a delayed, significant reduction in lymphatic transport after surgery which adversely affects the otherwise normally functioning collecting lymphatic vessel that was spared during the surgery. This has significant clinical applications, as it suggests that there could be a secondary delayed injury to a patient’s lymphatic pump that occurs after surgery - similar to what has been observed to occur in congestive heart failure due to remodeling after a cardiac event. As is also the case in heart failure, this delayed-onset reduction in lymphatic function is likely a multi-faceted problem driven by a multitude of inflammatory tissue repair processes and mechanically-mediated mechanisms. While the role of mechanical loading in guiding lymphatic development has recently gained traction^21–24^, the role of mechanical adaptation by lymphatics in the context of disease has been elusive. Additionally, the finding that lymphatic dysfunction persists, even when swelling resolves, suggests that therapies and interventions in the mouse that improve the reconnection of the lymphatic network where the wound is made, may not improve lymphatic pump capacity and are thus likely to be clinically unsuccessful in a patient where lymphatic pumping pressure is reduced.

This study also shows how the extent of swelling has a direct correlation with the dysfunction of the collecting lymphatic pump. This result is supported by measurements of packet transport and pumping pressure in mice with variation in gender, strain, and diet. Mice that swell significantly at any timepoint have sustained dysfunction in pumping pressure even as swelling returns close to baseline levels. Previous work combining computational and experimental data has shown that pumping pressure is a measurement of force generation of lymphatic muscle cells^25^. Low pumping pressure has also been described in humans with leg lymphedema, suggesting that a loss of collecting vessel pressure generation is correlated with the swelling experienced during lymphedema^26^. Given this physiological significance of pumping pressure, and the return of packet frequency measurements to baseline levels postsurgery, packet frequency alone is shown to be an inaccurate indicator of lymphatic pump function. Future work that studies lymphatic collecting vessels during disease progression cannot only rely on contraction frequency to describe lymphatic pump function.

Recent data from numerous animal and human studies of lymphedema have made it abundantly clear that inflammation is perhaps the most important factor driving lymphedema progression^3,7,27–29^, particularly as it relates to the changes that occur in the interstitium. Interactions between CD4^+^ T-cells and macrophages have been implicated in numerous studies in adversely driving lymphedema pathogenesis, and depletion of CD4^+^ cells ameliorates lymphedema in most mouse models^28–33^. It is thought that the mechanism of action is due to CD4^+^ cells driving adipose accumulation, tissue fibrosis, and abnormal lymphangiogenic responses in the skin. Ultimately, in a healthy lymphatic vasculature, transport is driven through the intrinsic pumping of the downstream collectors, and it is less clear whether these CD4^+^ driven changes in the skin are the driving cause of the disease or are symptomatic of a downstream failure in the collecting pump. Previous animal models have been incapable of addressing this, since fluid stasis is inevitable as every single lymphatic pathway draining the limb is surgically disrupted. This is contrary to clinical lymphedema where highly functional lymphatics are still intact and functioning after surgery^34,35^. Given that inflammatory cytokines have also been demonstrated to adversely affect collecting lymphatic pump function^36–38^, it is likely that the consequences of inflammatory driven lymphedema progression not only alter the interstitium, but severely compromise pump function and drainage by the collectors, furthering the fluid stagnation in the limb and resulting in a positive feedback-loop that drives disease progression.

One drawback of all mouse models of secondary lymphedema is that even if untreated, the swelling in the affected limb will resolve itself. Our data suggests that this is due to the compensatory ability of the initial lymphatic network to passively drain fluid from the interstitium through extrinsic mechanisms (e.g. lymph formation and elevated interstitial fluid pressure) that do not rely on strong intrinsic lymphatic contractility. The blood vasculature also may play a small role in compensatory drainage due to its ability to absorb excess fluid, but the lymphatics drain most of the interstitial fluid and protein that builds up during swelling. This compensatory effect in the mouse is likely significantly more effective at restoring fluid balance, as the adverse pressure gradients that the pump must overcome are much lower due to the minimal gravitational forces. This is contrary to a recent report in the mouse tail, which showed that the initial capillary network functionally regressed over the course of lymphedema development^39^. One fundamental difference in this study is that all of the lymphatic vessels downstream were severed as has been done in all other tail models of lymphedema, whereas in our study a collecting vessel was left intact. When considered together, these two studies suggest that the remodeling response to a complete downstream blockage (i.e. Gousopoulos model) is different than the remodeling response of a functional vessel that is overworked with an enhanced fluid demand (our model), even though tissue swelling and lymphatic hyperplasia are comparable between the two models. In clinical studies of lymphedema patients, it has been suggested that the latter is more likely to occur, as intact functional lymphatics are regularly identified in early stages of the disease. In fact some studies have suggested that an elevated fluid demand is present in at-risk patients, which would further stress the intact lymphatic vasculature after a surgical or radiation event^34,40^.

Our imaging approaches, which allow us to decouple intrinsic and extrinsic pumping mechanisms, demonstrate that the intrinsic pump is never restored even months after the swelling has gone down. Specifically, while the frequency of lymphatic contraction returns to normal, the ejection fraction of each contraction is reduced due to the loss in contractile amplitude, which results from a significant reduction in the pressure generating force of the lymphatic vessel chain. Additionally, quantifying the baseline function of the vessels prior to imaging clearly demonstrated that the severity of the pathogenesis depends on which lymphatic vessels are injured during surgery, as ligation of the dominant vessel resulted in significantly more swelling and a more severe functional deterioration in the intact vessel as compared to cases in which the non-dominant vessel was ligated.

The chronic reduction in lymphatic transport in the dominant ligation group through the three-month time point was an unexpected result. By this time the tissue did not exhibit any signs of swelling, and the ligation scar was no longer visible. Based upon visual inspection and volume measurements, the animals did not have lymphedema at this point. However, it is obvious from looking at the transport and pumping pressure data that the underlying lymphatic function remained severely compromised. These measured reductions in fluorescence intensity cannot be explained by changes in lymphatic vessel thickness, given that the change in thickness of the lymphatic vessel walls is minimal in comparison to the depth of these vessels below the surface of the skin. Changes in skin thickness could play a role in affecting fluorescence intensity, especially during initial swelling. However, at later timepoints swelling is reduced and significant changes in lymphatic pumping metrics are still observed, suggesting that the measured changes in function are real. This result is in line with those of Mendez *et al*. While this study did not directly measure lymphatic contractile function, they did observe a latent lymphatic insufficiency upon challenge with an inflammatory agent, that was not present in rats that had undergone an axillary lymph node dissection and subsequent remodeling in a rodent forelimb^3^. It is clear from our data that the lymphatic system sufficiently recovered to maintain fluid balance levels in the tail, which is why the tissue volume returned to normal. Despite the appearance of normal tissue, the reductions in transport and pumping pressure reveal that the vessels have remodeled such that they have lost a significant ability to pump, which would explain the inability to handle the elevated fluid loads associated with the immune challenge introduced by Mendez and colleagues. This is also in line with measurements of pumping pressure in human patients which have shown lower pumping pressures in patients with leg lymphedema^26^.

Clinical biopsies of advanced lymphedema patients have demonstrated alterations in smooth muscle cell content and phenotype in collecting vessels^41,42^, which could explain the observed decline in pumping pressure in our study. However, the vessels in the mouse were not sclerotic and actually had enlarged diameters which is different than what was observed in these human studies. Smooth muscle cell content and molecular composition was not investigated in this study, but future work in this regard could provide meaningful new insights. Alternatively, the reductions in transport and pumping pressure could have been caused by changes in valve function resulting in an inability to maintain unidirectional flow and decreasing pumping capacity. We have shown that the valve length to vessel diameter ratio is an important determinant of pump efficacy^43^ and thus an increase in vessel lumen diameter due to remodeling without a comparable increase in the valve length would reduce pump function. Under modest mechanical loading, the valves of isolated lymphatic vessel chains have been demonstrated to “lock” resulting in an inability to pump flow against an adverse pressure gradient^44^. Given that the behavior of lymphatic valves are highly sensitive to pressure^45^ and mutations affecting the proper formation of lymphatic valves is one of the most common causes of primary lymphedema^46,47^, it is reasonable to suspect non-optimal valve function may be contributing to the observed decreases in pumping capacity. However we were not able to measure lymphatic valve length or opening and closing characteristics in this study.

Histological analysis of collecting vessels dissected from the mouse tail suggest that both mechanisms are at play, as vessels distal to the wound exhibited significant dilation and qualitatively abnormal valve morphology. Interestingly, these morphological differences were not observed in the vessels of lymphedema animals located proximal to the wound. Given that proximal vessel function was also normal, it is highly likely that these morphological changes have a direct consequence on lymphatic function. Recent computational modeling by our group suggests that load-induced remodeling could result in morphologically-linked functional deterioration in lymphedema^48^. Furthermore, this result is in direct agreement with a recent study demonstrating that inflammatory-driven lymphangiogenesis mediated by macrophages and CD4^+^ T-cells significantly dilates collecting lymphatic vessels rendering them morphologically abnormal^31^. So while lymphangiogenesis is beneficial for repairing damaged lymphatic capillary networks, persistently high levels of lymphatic growth factors could be detrimental to intact collecting vessel function.

Analysis of the draining lymph nodes from the dominant ligation and sham groups revealed interesting trends regarding lymph transport routes. In the sham group the node draining the dominant vessel was larger and retained more fluorescence than the nondominant node, which again supports the theory of differential drainage patterns between the two vessels and agrees with a recent report regarding exosome transport to the node^49^. Not surprisingly, the lymphatic drainage patterns were altered after the dominant ligation to favor the non-dominant node, which drained the fully intact vessel. Interestingly, in the dominant group the nondominant node was approximately as large and retained approximately as much fluorescence as the dominant node of the sham group, perhaps suggesting some type of ideal physiological set point for lymph node size that is driven by the flow rate into the node. It is unclear what the ramifications of this change in drainage patterns might be, but it could have implications for immune response. Future investigation regarding the transport of immune cells, both through the local tissue environment and to the draining lymph nodes may help to provide insight on the link between lymphatic function and immune surveillance, especially in the context of reduced lymphatic function.

Obesity has long been demonstrated to be a primary clinical risk factor in the development of lymphedema. Motivated by this, several studies have demonstrated robust characterizations of the pathophysiology and the biological mechanisms that drive the pathogenesis of the disease during obesity^29,36^. However, despite their essential role in the active transport of lymph, the effects of obesity on the collecting lymphatic vessels have not been fully characterized during lymphedema. Specifically, no study has assessed collecting lymphatic pump performance over the course of lymphedema induction and resolution in the context of obesity. We have demonstrated that obesity adversely impacts lymphatic contractile activity and that this continues even after lymphedema is resolved. Further, we have demonstrated that pharmacological calcineurin inhibition, via tacrolimus, can protect against these functional declines. Even though treatment with tacrolimus following lymphatic ligation produced no significant reduction in tail swelling in obese mice, measurements of function in the intact vessel show that T-cell inhibition in the context of obesity improves lymphatic pump capacity. Without the single vessel ligation model, these changes in function would be missed and the benefit of tacrolimus treatment in this context would be unclear.

In this study we report for the first time that a secondary injury to an intact collecting lymphatic vessel occurs as the intact lymphatic vasculature remodels in response to an initial surgical insult. We were able to show the first definitive correlation between disease pathogenesis and the progressive loss of lymphatic pump function. We additionally showed that obesity exacerbated loss of lymphatic pump function in the intact collecting vessel following surgery, and tacrolimus treatment reversed these changes in lymphatic pump function. The novel lymphedema model presented here allows for analysis of collecting vessel remodeling and lymphatic functional changes following lymphatic damage that were unattainable with existing lymphedema models. Future work that combines this novel animal model with functional imaging and lymphatic pumping pressure measurements could fully describe the collecting vessel hyperplasia observed during lymphedema progression and could elucidate the mechanism of this remodeling. The model could also be used to screen for molecular targets that minimize this collecting vessel hyperplasia, leading to new therapeutic options for treating and preventing post-surgical lymphedema.

## Materials and Methods

### Surgical model and time course measurements

The rodent tail model of lymphedema has been used extensively in previous studies^8,9,27,50^. Generally, a dermal-layer incision is made spanning the entire circumference of the tail such that all collecting and capillary lymphatics are severed. This technique produces a well-documented swelling cascade that closely resembles the progression of lymphedema, at least in the dermal layer.

In an attempt to study the role of collecting lymphatic vessel function in lymphedema onset and progression, we modified the conventional tail lymphedema model. Specifically, we altered the dermal incision such that it spanned nearly the entire circumference of the tail with the exception of allowing one collector to remain intact (Fig. 1A–C).

Previously, our group has characterized the lymphatic physiology of the tail as having a dominant and a nondominant collecting trunk^19^. Given this, we hypothesized that injury to the dominant collecting vessel would produce greater swelling due to the increased reliance on this vessel for fluid transport from the tail. Thus, the present study included three experimental groups: (1) a vessel ligation group in which the dominant vessel was ligated (n = 4), (2) a vessel ligation group in which the nondominant vessel was ligated (n = 4), and (3) a sham control group in which both collecting vessels were left intact (n = 4). Eight-week-old female balb-c mice (Charles River Laboratories, Wilmington, MA) were used for this study according to procedures approved by the Georgia Institute of Technology IACUC Review Board. All animals were first anesthetized using inhaled isoflurane (5% induction, 2% maintenance). All three groups received incisions 1 cm from the base of the animal spanning 80–90% of the circumference of the tail with particular care to standardize the incisions as much as possible. In the vessel ligation groups, the incision severed one collecting vessel and left one intact, while in the sham control group the incision severed a substantial percentage of the lymphatic capillary network, but none of the collectors were severed. All incisions were cauterized to prevent bleeding and fluid leakage. After the surgical procedures, both collecting vessels were checked with NIR imaging to ensure they were either severed or remained intact as appropriate. Animals in which vessels were improperly ligated or in which blood vessels were ligated were excluded from the study.

Baseline metrics were collected in all groups prior to surgery. NIR functional metrics were again measured after surgery at days 2 and 4, weeks 1, 2, and 3, and month 3. Tail measurements were taken every two days via two-dimensional projected area to track swelling progression. Lymphatic pumping pressure measurements were taken before surgery and at 2 day, 4 day, 1 week, and 3 month time points. All animals were euthanized after 3 months and excised collecting vessels were harvested for analysis.

For the other group of mice included with this study, NIR functional metrics, tail swelling measurements, and pumping pressure were measured after surgery at 1, 2, 4, 6, 8, and 10 weeks post-surgery.

### Immunohistochemistry

Tail sections were harvested at two weeks following lymphatic injury in the dominant vessel ligation group. Tail sections were also harvested at two weeks in mice that underwent double vesssel ligation. Tissues were harvested distal and proximal to the site of injury. These tissues were fixed in 10% neutral-buffered formalin, embedded in paraffin, and 5 µm sections were sliced for immunohistochemical analysis. For immunofluorescent staining, sections were treated with sodium citrate in a 90 °C water bath for antigen retrieval. Non-specific binding was blocked with 20% goat serum, 1% bovine serum albumin (BSA) and 79% PBS for one hour at room temperature. Sections were then incubated overnight at 4 °C with a hamster monoclonal anti-podoplanin (1:100, ab11936) primary antibody from Abcam (Cambridge, MA). Sections were then incubated with a corresponding secondary antibody (1:200, A21110) from Thermo Fisher Scientific for four hours at room temperature. A Zeiss LSM 700 confocal microscope was used to image slides after staining, and analysis was performed on high-powered sections (20×) with at least 6 high-powered fields (hpf) per animal.

### NIR functional imaging

NIR lymphatic imaging was performed according to previously published methods^51^. Briefly, 10 μL of LI-COR IRDye 800CW PEG (LI-COR Biosciences, Nebraska, USA) was injected intradermally into the tip of the tail for fluorescence imaging (the fluorophore was reconstituted according to manufacturer instructions for lymphatic imaging). An injection volume of 10 μL was chosen based upon past success in rodent models, so as to not overload the lymphatics with unnecessary fluid volume while at the same time providing enough tracer for sufficient detection^19^. The injection was given at an entry angle of approximately 10 degrees to an approximate depth of 1 mm to specifically target the lymphatic vasculature. Care was taken to position the injection as close to the midline of the tail as possible to avoid favoring one collecting vessel over the other. Images were taken with a customized imaging system consisting of a Shutter Instruments Lambda LS Xenon arc lamp, an Olympus MVX-ZB10 microscope, a 769 nm bandpass excitation filter (49 nm full-width half maximum, FWHM), an 832 nm bandpass emission filter (45 nm FWHM), and an 801.5 nm longpass dichroic mirror. Images were acquired with a Photometrics Evolve Delta 512 EM-CCD. The field of view was centered on the mouse’s tail 7 cm downstream (towards the base of the tail) from the injection site at the tip of tail. This location ensured that only the downstream collecting lymphatics would be visualized. The small volume of fluid injection and the use of NIR to enhance tissue penetration ensures that only fluorescence in the deeper collecting lymphatics is visible downstream of the injection site. The animals were imaged continuously from the time of injection until 20 minutes post-injection with a 50 ms exposure time and a frame rate of 10 fps. Analysis of NIR functional metrics was performed during the steady-state period ranging from 5–20 minutes after injection, as defined previously^19^.

NIR functional measurements included a combination of previously reported metrics and newly developed metrics for this model. Packet frequency, emptying rate, and pumping pressure were measured and recorded as previously published^51^. To measure lymphatic pumping pressure, a pressure cuff was placed around the tail, 6 cm from the tip of the tail. The pressure was increased to 80 mmHg, held for 5 minutes, decreased down to 55 mmHg, and lowered to 0 mmHg in increments of 2.5 mmHg for 5 seconds at each pressure. Fluorescence intensity in the collecting vessel distal to the cuff was measured, and lymphatic pumping pressure was quantified as the pressure in the cuff when intensity was halfway between its minimum and maximum value as flow resumed past the cuff. Transport time was used to determine the identity of the dominant and nondominant collector, but was not used during time-course analysis of lymphatic function. Packet velocity was also not used for functional analysis because the dynamics of mouse contractile function, especially in the diseased cases, prevented reliable velocity measurements.

### Characterization of NIR fluorescent packets in isolated vessels and *in vivo*

In order to better characterize NIR functional measurements of lymphatic transport *in vivo*, two experimental setups were used to assess fluorescent packets, as they appear in collecting vessels *in vivo*. In the first setup, mesenteric collecting vessels were excised from Sprague Dawley rats (Charles River Laboratories, Wilmington, MA) and were cannulated and pressurized in a living system chamber according to previously published methods^52^. Rat mesenteric vessels were chosen over mice due to the difficulty in the preparation to isolate functional mouse tail lymphatics. A solution containing the commercially available fluorophore LI-COR IRDYE 800CW PEG (LI-COR Biosciences, Nebraska, USA) diluted to 10% of the manufacturer’s recommended concentration for lymphatic imaging was flowed through the vessel. Images were taken in brightfield to record vessel contraction and in NIR to observe transport of the dye through the vessels (Fig. 2A). The purpose was to assess the relationship between the appearance of packets and contractions of the vessel.

The second experimental setup to characterize packets utilized *in vivo* NIR imaging with the skin removed. *In vivo* NIR imaging was performed as detailed above with the exception that the imaging was performed in the forelimb and the injection was given in the footpad. Once fluorescence arrived in the collecting vessels downstream from the injection site, images were taken with the skin on and with the skin off to determine the effect of skin on the appearance of packets (Fig. 2B).

### Development of new NIR metrics

A new NIR functional metric was developed called Lymphatic Fluorescence Transport. Packets were detected by first identifying peaks in the fluorescence signal and then identifying the time points at which troughs on either side of the peak occurred (Fig. 2B). The fluorescence signal was then integrated across each packet and summed over the length of the data set. Lymphatic fluorescence transport was then calculated as the time-normalized sum of the packet integrals such that:

$$Lymphatic\,Fluorescence\,Transport=\,\frac{{\sum }_{i=1}^{n}\,{\int }_{t\,{\rm{\min }}\,i}^{t\,{\rm{\max }}\,i}\,f(t)dt}{time}$$

where *n* is the number of packets in the data set and *f*(*t*) is the fluorescence signal within each packet region (between the minimum and maximum time points corresponding to each packet). This metric represents the cumulative transport of fluorescence through the vessel normalized by time and was used to assess overall lymphatic transport capacity. Average packet amplitude was also measured and recorded to assess contractile function of the vessels.

In order to quantify the relative amounts of initial lymphatic vessel recruitment in fluid transport compared to collecting vessel transport across the different experimental conditions, we utilized a new metric called fluorescence area. Fluorescence area was measured during the 10 min imaging segment following fluorophore injection by converting the grayscale image to binary using cluster-based thresholding. The total area of positive pixels above this threshold was calculated and then normalized by the total area of the tails. This yielded the percentage of the tail positive for fluorescence as a function of time.

### High-fat diet obesity model

8-Week-old male C57BL6J mice were placed on a high fat diet (n = 11) (60% kcal fat, TestDiet 58Y1, W.F. Fisher & Son, Sommerville, NJ) or normal, uncontrolled chow diet (n = 8) (13% kcal fat, Purina PicoLab Rodent Diet 20, W.F. Fisher & Son, Sommerville, NJ) for 20 weeks. Mouse bodyweight was measured using a digital scale. Mice were housed one per cage in a temperature and humidity controlled (21 ± 1 °C) vivarium on a 12-hr light, 12-hr dark cycle. Food and water were available ad libitum. The goal of this study was to compare the lymphedema response in obesity to the normal observed lymphedema response, so a low-fat diet was not used as a control.

### Topical tacrolimus treatment

0.1% (wt/wt) tacrolimus ointment was made using tacrolimus powder and a base. 10 mg of tacrolimus powder (FK-506, Fisher Scientific, Pittsburgh, PA) was levigated in 1 mL of anhydrous glycerine (Sigma Aldrich, St. Louis, MO) with a glass stir rod. Roughly 100 g of Aquaphor (Beiersdorf Inc., Wilton, CT) was melted at 40 °C until liquefied. The tacrolimus and glycerine mixture was geometrically diluted in this liquefied Aquaphor base to a final weight of 100 g, stirring thoroughly in between dilutions to ensure even distribution of tacrolimus in the suspension. The ointment was then sealed in an air tight plastic jar and stored at room temperature in a dark place until use. Topical tacrolimus treatment of a subset of obese mice (n = 5) began 14 days after the induction of lymphedema via the ligation surgery, immediately after the 14-day NIR imaging time point. Roughly 100 mg of tacrolimus ointment was applied in an even coat to the entirety of the tails twice daily until the end of the 70-day study.

### Statistical analysis

Tail swelling and lymphatic function metrics were compared between the two ligation groups as well as between each ligation group and the sham group via two-way ANOVA with Tukey and Bonferroni multiple comparison corrections as appropriate. The correlation between tail swelling and lymphatic packet transport and packet frequency was calculated using linear regression, and an F-score obtained from linear regression was used to determine significance. Unpaired t-tests were used for comparisons between two groups. Multiple t-tests were used to compare swelling between groups over time, with the Holm-Sidak method used to correct for multiple comparisons. Alpha was equal to 0.05 for all statistical analysis^53–56^.
